# Supplementary material for: Filbertone Reduces Senescence in C2C12 Myotubes Treated with Doxorubicin or H2O2 through MuRF1 and Myogenin
Source: Nutrients. 2024 Sep 19;16(18):3177. doi: 10.3390/nu16183177 (PMC11434963; doi:10.3390/nu16183177)
Supplement: Supplementary file 1 [file nutrients-16-03177-s001.zip › nutrients-3151501-supplementary.pdf]

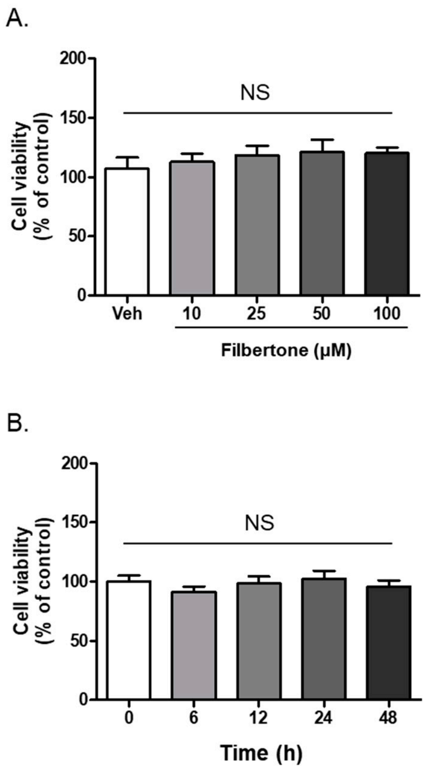

Figure S1. Cytotoxic effects of filbertone on C_2C_{12} myotubes. (A) Cell viability was determined by MTT assay following treatment with filbertone (10, 25, 50, 100μM) for 24h (n=5 per group). All results are expressed mean ± SD. NS means not significant compared to Veh by one-way ANOVA with Tukey multiple comparison post-hoc test. (B) Cell viability was determined by MTT assay following incubation with filbertone (100μM) for the indicated time (n=5 per group). All results are expressed mean ± SD. NS means not significant compared to 0h by one-way ANOVA with Tukey multiple comparison post-hoc test.

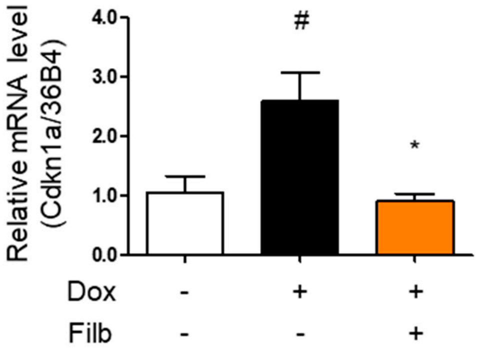

Figure S2. Effect of Filbertone on the gene expression of p21 (Cdkn1a) in senescence-induced C2C12 Myotubes. Gene expression of Cdkn1a was measured by qRT-PCR in myotubes following incubation with doxorubicin (1μM for 6h) in the presence of filbertone (100μM for 24h). The levels of Cdkn1a gene expression were normalized to the 36b4 levels in each sample (n = 3 per group). Results are expressed as mean ± SD. # $p<0.05$ compared to Veh; * $p<0.05$ compared to Dox(only) by one-way ANOVA with Tukey multiple comparison post-hoc test.

Table S1. Mouse primer sequence for qRT-PCR.

Mouse Gene	Forward (5' to 3')	Reverse (5' to 3')
Trp53	CTCCGAAGACTGGATGACTG	ACAGATCGTCCATGCAGTGAG
Cdkn1a (p21)	TGTCTTGCACTCTGGTGTCTG	CAATCTGCGCTTGGAGTGAT
Myogenin	TGCCCAGTGAATGCAACTCC	TCCACCGTGATGCTGTCCA
MuRF1	TGTCTCACGTGTGAGGTGCCTA	CACCAGCATGGAGATGCAGTTAC
Rplp0 (36B4)	TGGAAGTCCAACTACTTCCTCAA	ATCTGCTGCATCTGCTTGGAG
